# Changing epidemiology and etiology of hepatocellular carcinoma in Europe: implications for clinical practice and health policy

**DOI:** 10.1016/j.lanepe.2026.101837

**Published:** 2026-09-03

**Authors:** Bernhard Scheiner, Jeroen Dekervel, Fabio Piscaglia, Alessandro Vitale, Loreta Anesti Kondili, Bogdan Procopet, Jeffrey V. Lazarus, Maria Buti, Shira Zelber-Sagi, Matthias Pinter

**Affiliations:** a Division of Gastroenterology and Hepatology, Department of Medicine III, Medical University of Vienna, Vienna, Austria; b Digestive Oncology, Department of Gastroenterology and Hepatology, University Hospitals Leuven, Herestraat, Leuven, Belgium; c Division of Internal Medicine, Hepatobiliary and Immunoallergic Diseases, IRCCS Azienda Ospedaliero-Universitaria di Bologna, Italy; d Department of Medical and Surgical Sciences, University of Bologna; e Department of Surgical Oncological and Gastroenterological Sciences, Padova University, Padova, Italy; f National Center for Global Health, Istituto Superiore di Sanità, Rome, Italy; g Saint Camillus International University of Health Sciences, Rome, Italy; h 3rd Medical Clinic – Gastroenterology Department, Iuliu Hațieganu University of Medicine and Pharmacy, 400012, Cluj-Napoca, Romania; i Hepatology Department, Octavian Fodor Regional Institute of Gastroenterology and Hepatology, 400158, Cluj-Napoca, Romania; j CUNY, Graduate School of Public Health and Health Policy, New York, NY, USA; k Barcelona Institute for Global Health (ISGlobal), University of Barcelona, Barcelona, Spain; l Division of Gastroenterology and Hepatology, Department of Medicine, New York-Presbyterian Hospital/Weill Cornell Medical Center, New York, NY, USA; m Liver Unit, Hospital Univesitari Vall d'Hebron and CIBERHED del Instituto Carlos III, Barcelona, Spain; n School of Public Health, Faculty of Social Welfare and Health Sciences, University of Haifa, Haifa, Israel; o The Tel-Aviv Medical Center, Department of Gastroenterology and Hepatology, Tel-Aviv Medical Center, Tel-Aviv, Israel

**Keywords:** Alcohol, Liver cancer, MASLD, Public health, Viral hepatitis, Incidence

## Abstract

Primary liver cancer, of which hepatocellular carcinoma (HCC) represents the vast majority, is on the rise globally. Despite an expected decrease in viral hepatitis-related HCCs, the number of new cases and deaths from liver cancer is predicted to increase by over 30% in 2050 in Europe. This is largely driven by an increase in metabolic dysfunction-associated steatotic liver disease-related HCC and an aging population. Since HCC usually develops in patients with underlying chronic liver disease, over half of all cases appear to be preventable by targeting modifiable risk factors. This series paper summarizes epidemiological and etiological trends of HCC in Europe and their implications for the clinical management of HCC across different disease stages. We discuss public health initiatives and policies to counteract the rising incidence of HCC by focusing on prevention, and highlight additional benefits of etiological treatment for the management and prognosis of patients with HCC.

**Key messages**

- HCC incidence in Europe is rising due to an increase in HCC related to MASLD, persistently high alcohol consumption, and an aging population. Meanwhile viral hepatitis-related HCC is declining in several countries.
- Optimal screening and treatment strategies remain challenging given the increasing proportion of non-cirrhotic HCC.
- Etiological therapy may prevent HCC development, improve liver function and even lead to recompensation. This allows patients who were previously ineligible due to poor liver status to become candidates for anti-tumor treatments.
- More than half of HCC cases are potentially preventable, yet preventive measures remain critically under-implemented at a population level.

## Introduction

Primary liver cancer, of which hepatocellular carcinoma (HCC) accounts for 75%–85% of the cases, represents the third leading cause of cancer-associated mortality, resulting in almost 900,000 deaths globally in 2022.^1^ While the majority of incident liver cancer cases are observed in Africa and Asia, around 62,000 new cases were diagnosed and around 54,000 patients died from liver cancer in Europe in 2022.^2^ In contrast to other cancer types, the number of cases of liver cancer is predicted to increase globally, mainly as a result of an aging and growing population and an increasing number of patients with metabolic liver diseases.^3^ The Lancet Commission on liver cancer, a panel of global clinical and public health experts, estimated that at least 60% of liver cancers, particularly HCC, are preventable by targeting modifiable risk factors (e.g., viral hepatitis, alcohol and metabolic comorbidities).^4^

While effective antiviral therapies are widely available in Europe and some countries are on track to achieve the World Health Organization (WHO) target of hepatitis C virus (HCV) elimination by 2030, substantial regional disparities in screening and micro-elimination programmes persist, limiting the impact of these efforts on reducing HCC incidence.^5^ Furthermore, the global rise in alcohol-related liver disease and metabolic dysfunction-associated steatotic liver disease (MASLD; previously called non-alcoholic fatty liver disease (NAFLD)) outweighs the reduction in viral hepatitis-related HCC cases.^4^^,^^6^ This is associated with a significant change towards a higher number of cases with non-cirrhotic HCC associated with MASLD, which are frequently diagnosed at later stages as structured screening programs in non-cirrhotic patients have not been established yet.^7^

In this series paper, we summarize epidemiological trends of HCC, their underlying etiological causes, as well as their implications for the management of HCC in Europe. Moreover, we discuss public health initiatives and policies to counteract the rising incidence of HCC by focusing on prevention.

## Epidemiology of HCC in Europe

Global age-standardized liver cancer incidence rates are highly region-specific and vary between 2.6 and 10.1 per 100,000, with a strong male predominance (Fig. 1). Not only globally, but even in Europe, age-standardized incidence rates vary significantly, with the highest rates observed in Southern Europe (3.1 and 9.7 per 100,000 in females and males, respectively) and the lowest rates in parts of Northern and Eastern Europe (2.5 and 6.6 per 100,000 in females and males, respectively).^1^^,^^8^ Despite growing awareness and health system improvements, liver cancer incidence (Fig. 1) and mortality (Fig. 2) in Europe have significantly increased by an average annual percentage change (AAPC) of 1.05% from 1990 to 2021.^9^ These numbers are well above the reported global AAPC of 0.12%,^9^ constituting liver cancer development in Europe as a real problem.^10^

**Fig. 1:**
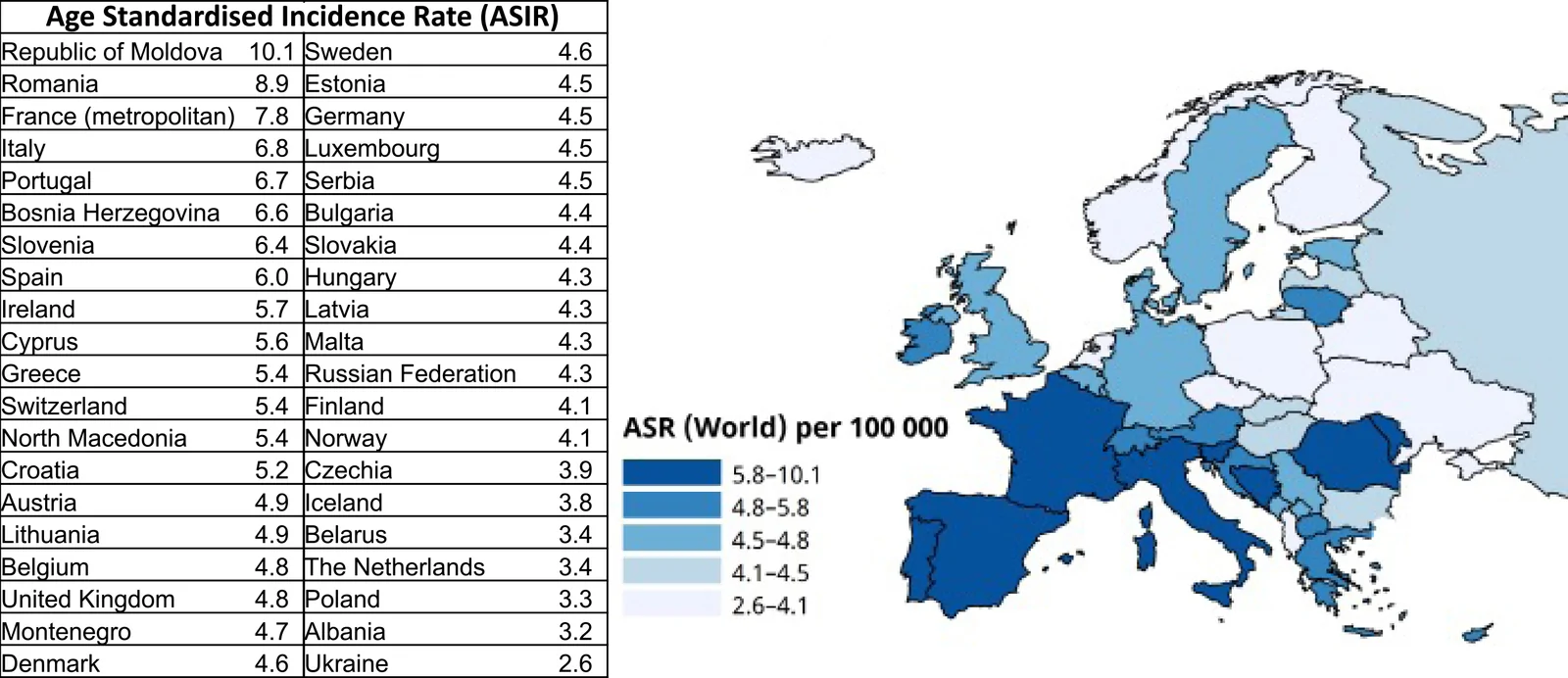
**Incidence (age-standardized rate [ASR] per 100,000) of liver cancer in Europe in 2024 (Data extracted with permission from Ferlay J. et al. Global Cancer Observatory: Cancer Today (version 1.1). 2024****^8^****)**.

**Fig. 2:**
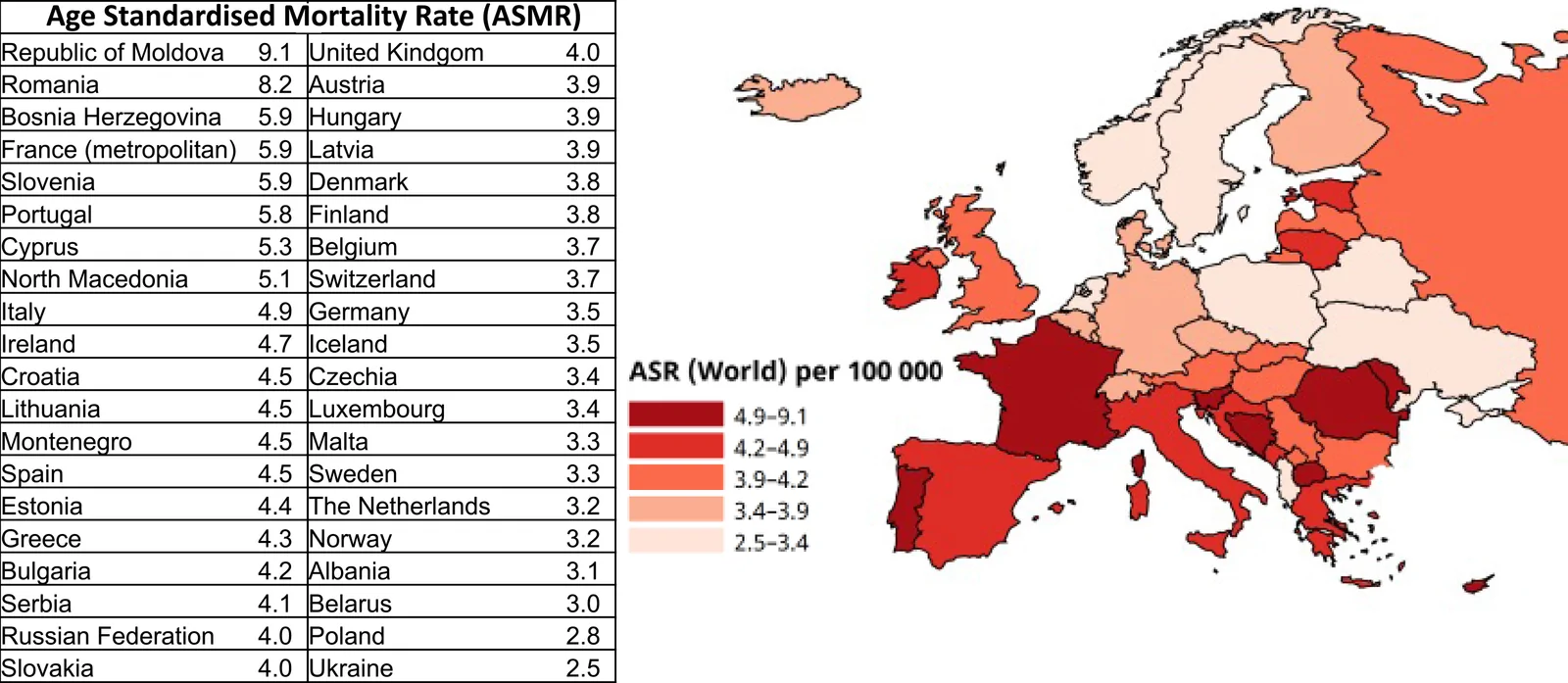
**Mortality (age-standardized rate [ASR] per 100,000) of liver cancer in Europe in 2024 (Data extracted with permission from Ferlay J. et al. Global Cancer Observatory: Cancer Today (version 1.1). 2024****^8^****)**.

### Epidemiological trends in Europe

As the majority of incident HCC cases develop in the setting of advanced chronic liver disease,^11^ the epidemiology of HCC in Europe is strongly correlated with changes in the prevalence and treatment of the underlying liver diseases.^4^ In the absence of harmonized pan-European, population-based registry data with detailed information on the underlying etiology of liver disease, this review utilises Global Burden of Disease (GBD) estimates to provide a Europe-wide overview on etiological trends.^3^ Additionally, we discuss the available direct evidence including three population-wide studies from Sweden, the Netherlands and Madeira^12^, ^13^, ^14^ as well as several tertiary referral cohorts and single-center reports from different European countries.^12^^,^^14^, ^15^, ^16^, ^17^, ^18^, ^19^, ^20^, ^21^, ^22^ Importantly, Global Burden of Disease (GBD) estimates are indirect and assumption-dependent, and should not be interpreted on the same evidence level as primary population-based cohorts.^3^

GBD estimates suggest that chronic HCV infection accounted for ∼41% of HCC cases in Europe in 2019.^23^ These proportions are expected to decline due to the impact of highly effective direct-acting antivirals (DAA) and local micro- and macro-elimination programs.^24^ GBD also suggests modest declines in HBV-associated HCC as a result of childhood HBV vaccination programs that have been established in 27/30 EU/European Economic Area (EEA) countries.^25^ While those HBV elimination programs are expected to further reduce the number of HBV-associated HCC cases in the future, concomitant MASLD increases HCC risk in HBV-positive individuals.^26^ Furthermore, a significant proportion of patients with chronic HBV and HCV infection remains undiagnosed, resulting in a substantial pool of patients at risk of progressing to liver cirrhosis and liver cancer.^27^ Hepatitis D virus (HDV), a rare but highly aggressive form of chronic viral hepatitis, also persists in hotspots across Eastern Europe and the Mediterranean basin.^28^ HDV co-infection accelerates progression to cirrhosis and substantially increases HCC risk,^28^ underscoring the need for systematic HDV testing of all individuals with chronic HBV infection.

GBD data also indicate that alcohol consumption-associated HCC incidence remains very high in Europe and accounts for around 35–50% of overall HCC cases—the highest proportion worldwide.^14^^,^^29^

While robust data on absolute changes in incident cases are scarce, the most worrisome proportional changes in HCC epidemiology in Europe are characterized by a rapid increase in HCC cases due to MASLD and metabolic dysfunction-associated steatohepatitis (MASH; previously called non-alcoholic steatohepatitis (NASH)). The strongest increases have been projected in Western, Southern and Eastern Europe.^3^^,^^9^^,^^30^ Importantly, these projections primarily reflect changes in etiologic fractions and modeled incidence rates and should therefore not be interpreted as direct measurements of the current population risk.

Population-based registries and tertiary referral cohorts support the GBD-derived trends, although the exact etiologic proportions often differ between model-based estimates and empirical data. While strong declines in the proportion of HCV-associated HCC have been reported in studies from Spain and Italy,^17^^,^^18^ no clear declines have recently been observed in reports from Sweden, where every fifth HCC was related to HCV.^12^ Despite a persistently high HCV burden in Romania,^15^ HCV elimination efforts have already translated into a strong decline in the proportion of HCV-associated liver transplantations in the European region.^31^

Direct European data confirm modest declines in HBV-related HCC with relatively low levels in Northern and some Southern European countries and slight decreases in HBV-associated liver transplantations.^13^^,^^18^^,^^31^ Nevertheless, HBV-associated HCC remains a relevant concern in Madeira, Romania and Slovakia.^14^, ^15^, ^16^

Considering the overall case load, alcohol-related liver disease remains the leading etiology of liver disease in the majority of European countries^24^ with age-standardized alcohol-related HCC incidence rates remaining relatively stable over the last decade.^29^ According to data from several European countries such as France, Austria, Germany, Slovakia and Madeira, up to every second HCC case is related to harmful alcohol abuse.^14^^,^^16^^,^^19^, ^20^, ^21^

In line with GBD estimates, metabolic liver disease associated HCC is strongly increasing across Europe and has even become the leading cause of HCC in some European countries, such as Sweden, even surpassing viral hepatitis.^12^ This trend is not only observed in countries where MASLD-associated HCC has always been a relevant problem, but also in countries such as Spain, where the proportion of HCC related to metabolic syndrome has been low. Longitudinal data from a Spanish multicenter registry found that the proportion of HCC attributed to liver disease related to the metabolic syndrome increased from 4.9% in 2008/2009 to 24% in 2022/2023 with the actual case number increasing from 21 to 118 in the participating centers over the same period.^18^ Concomitantly, the rate of non-cirrhotic HCC is also increasing.^18^^,^^22^

### European trends in a global context

In line with the European situation, marked regional differences in HCC etiology are also observed outside of Europe. In the United States, HCV historically dominated the HCC landscape, but following the widespread use of DAA treatment its contribution to HCC-related liver transplantation fell by nearly half while at the same time, there has been a marked shift toward alcohol-related and metabolic liver disease.^32^ In South America, MASLD/MASH-related HCC has quadrupled over the last decade and already represents the most important underlying cause of liver disease in patients with HCC accounting for more than one third of cases overall.^33^ In contrast, HBV remains the predominant etiology of liver disease in most Asian countries with the exception of Japan, where HCV dominates and the proportion of MASLD-related HCC is rising, mirroring the global trend towards metabolic liver disease.^34^

### Population mobility, viral hepatitis and HCC risk

Population mobility contributes to Europe's changing liver disease epidemiology, but migration itself is not an intrinsic HCC risk factor. Risk is shaped by country and region of birth, age and route of viral hepatitis acquisition, infection duration, metabolic exposures, and access to prevention and care.^6^ As of January 2025, 46.7 million people (10.4% of the EU population) were born outside the EU, including nearly 30 million non-EU citizens.^35^ Within this demographic context, an earlier ECDC epidemiological assessment estimated that migrants account for 25% of chronic HBV and 14% of chronic HCV infections in the EU/EEA,^36^ highlighting the substantial relative burden of chronic HBV among migrant populations. A recent modeling study estimated that, in 2024, 1.73 million people were living with chronic HBV and 1.03 million with chronic HCV in the EU-27.^37^ This substantial burden of chronic viral hepatitis contributes to population-level HCC risk, which is further influenced by age and route of acquisition, duration of infection, fibrosis stage, metabolic cofactors, and access to diagnosis and treatment. People born in regions in sub-Saharan Africa and Asia, where HBV is highly endemic are more likely to have acquired infection perinatally or during early childhood, resulting in prolonged chronic infection and an increased lifetime risk of cirrhosis and HCC.^38^ In Europe, HCC mortality has been reported to be four-to-five times higher in some foreign-born groups than in native-born populations and approximately threefold higher among foreign-born women.^38^ In a multicentre European cohort of sub-Saharan African and Afro-Surinamese individuals with chronic HBV, the 10-year cumulative HCC incidence was 2.4% overall but reached 12.1% among those with advanced fibrosis.^39^ In this context, the relative contribution of underlying liver diseases differs across populations. While MASLD and alcohol-related liver disease account for an increasing proportion of HCC, chronic viral hepatitis remains a major cause among people born in countries where HBV or HCV is highly endemic.^30^^,^^40^

Metabolic exposures after relocation may further modify HCC risk. Evidence from an umbrella review suggests that longer duration of residence in high-income countries is associated with increasing obesity risk,^41^ although Europe-specific longitudinal evidence remains limited. Dietary change associated with acculturation, together with food insecurity and socioeconomic disadvantage, may further increase metabolic risk.^41^ A European systematic review and meta-analysis reported higher odds of type 2 diabetes among South Asian (odds ratio [OR] 3.7), and Middle East and North African populations (OR 2.7) than among host European populations.^42^ When the resulting metabolic dysfunction coexists with chronic viral hepatitis, viral and dysmetabolic pathways may interact to amplify HCC risk.^4^

Structural barriers to screening, treatment, and HCC surveillance remain particularly important for refugees, asylum seekers, and undocumented people. Legal precarity, language barriers, stigma, and limited culturally adapted services may contribute to delayed HCC diagnosis and reduced access to care.^6^ Swedish cohort data showed that HCC incidence exceeded the threshold considered sufficient to justify surveillance in Asian men with chronic HBV aged 40–49 years and in men from other regions of origin aged 50–59 years. After excluding individuals with cirrhosis or previous antiviral treatment, HCC incidence remained above this threshold in Asian men aged 40–49 years.^43^ These findings support equitable HBV/HCV testing, universal access to antiviral treatment irrespective of legal status, risk-based HCC surveillance, particularly for those with cirrhosis or diabetes, and culturally adapted metabolic prevention within comprehensive care strategies as core components of universal health coverage.^6^^,^^44^

## Implications of etiological trends on management of HCC

The evolving epidemiology of HCC in Europe has important implications for clinical management including the identification of populations eligible for surveillance and the management of early, intermediate and advanced stage HCC.

### Defining populations at risk for HCC surveillance

The exact target population for HCC surveillance as well as the optimal surveillance modalities are increasingly being questioned in the context of the evolving epidemiology. In the past, surveillance was universally recommended for patients with established cirrhosis, excluding those with severely impaired liver function or comorbidities precluding treatment.^11^ This approach was justified because HCC was strongly associated with cirrhosis, mainly driven by chronic HCV infection and alcohol-related liver disease. However, up to 40% of MASLD-related HCCs develop in the absence of cirrhosis.^45^ Panel 1 summarizes populations at risk for HCC, together with proposed surveillance strategies and their limitations.

Panel 1 **Populations at risk for HCC and proposed surveillance strategies.**

Patients with MASLD and cirrhosis should be submitted to surveillance, provided they are theoretically eligible to receive effective treatment, but it is largely unclear which non-cirrhotic MASLD patients should undergo surveillance. Indeed, surveillance is generally not recommended for patients who have not reached the stage of cirrhosis yet, since evidence of cost-effectiveness is lacking. Several predictive models have attempted to identify high-risk subgroups^46^ and an individualized consideration whether or not to start surveillance could be applied to patients without cirrhosis, but with severe fibrosis (usually those with liver stiffness >10 kPa) and additional risk factors for HCC development such as diabetes mellitus, older age, male gender, and decreased platelet count.^47^ Surveillance could become reasonable under these circumstances. However, even in MASLD patients with advanced fibrosis (F3), annual HCC incidence often does not consistently exceed the 0.5–0.7% yearly threshold generally considered necessary to justify cost-effective surveillance.^48^

A second major issue concerns patients with cured HCV cirrhosis. Recent cost-effectiveness analyses suggest that surveillance may be discontinued if annual HCC incidence falls below 0.7%,^49^ but current studies have not reliably identified which patients fall below this risk level.^50^ Moreover, risk factors such as aging or superimposed MASLD may accumulate over time, making it difficult to determine when surveillance can safely be stopped or should be resumed. Patients with effectively treated HBV but concomitant cirrhosis should continue surveillance indefinitely.^11^ In non-cirrhotic HBV patients, surveillance initiation is largely guided by validated risk scores.^51^ Conversely, in patients with cured HCV without cirrhosis, surveillance is generally not recommended.^11^

Ultrasound every six months, with or without alpha-fetoprotein (AFP), remains the standard surveillance modality. Reintroduction of AFP has gained renewed relevance because viral inflammation is now largely controlled, reducing false-positive rates.^11^ Importantly, ultrasound reports should document liver explorability in a standardized manner, such as the Liver Imaging Reporting and Data System (LI-RADS) visualization score.^52^ Patients with poor visualization—common in those living with obesity and/or MASLD—may require alternative imaging modalities such as abbreviated magnetic resonance imaging (MRI).^53^

### Management of early-stage HCC

The etiological shift towards MASLD has multifaceted implications for the management of patients with HCC who are surgical candidates.^11^ Patients with MASLD-related HCC frequently present with complications of the metabolic syndrome, which collectively increase perioperative risk and have been identified as independent predictors of major complications after hepatectomy.^54^ Therefore, pre-operative assessment should systematically address the metabolic and cardiovascular conditions most consistently associated with postoperative morbidity and graft loss^55^: glycemic control, since diabetes predicts both waitlist dropout and post-transplant graft failure; cardiovascular risk stratification, given that major adverse cardiac events are a leading cause of early post-transplant mortality in this population; and body composition, since sarcopenic obesity rather than body mass index alone drives adverse surgical outcomes.^56^ Structured multidisciplinary prehabilitation pathways integrating hepatology, surgery, cardiology, diabetology, and nutrition may allow modifiable risk to be reduced before surgery and candidacy to be assessed more accurately, with direct implications for transplant prioritization policies.^57^ Moreover, minimally invasive liver surgery (MILS) significantly reduced major complications and post-hepatectomy liver failure in patients with metabolic syndrome compared with open surgery.^54^^,^^58^

MASLD-related HCC in a non-cirrhotic liver with preserved hepatic functional reserve may favor candidacy for resection.^45^ However, transplantation in candidates with MASLD-related cirrhosis introduces specific challenges. Patients with MASLD without HCC had a significantly increased risk of waitlist death/dropout driven by liver-related events and a lower probability of reaching transplantation.^59^ US registry data confirmed that MASLD and alcohol-related liver disease had the highest waitlist dropout rates among HCC candidates, with pre-transplant diabetes emerging as a key predictor of both waitlist dropout and post-transplant graft failure.^60^ Nevertheless, a recent meta-analysis found comparable post-transplant overall survival and disease-free survival between MASLD-related HCC and other aetiologies, suggesting that carefully selected MASLD patients should not be denied transplantation.^61^

Despite being more often diagnosed at later stages—owing to the lack of systematic surveillance in the non-cirrhotic population—MASLD-related HCC appears to have a less aggressive biological phenotype with better long-term overall survival and fewer recurrences after liver resection for metabolic syndrome-related HCC than for HCV-related HCC.^62^^,^^63^ However, recurrence and *de novo* carcinogenesis remain substantial concerns, especially when high-risk lesions are considered for resection. In this regard, the emerging landscape of perioperative immunotherapy is poised to reshape the management of resectable HCC. The CARES-009 phase 2/3 randomized controlled trial (RCT) showed that perioperative camrelizumab plus rivoceranib significantly improved event-free survival compared with surgery alone in patients at intermediate-to-high risk of recurrence.^64^ However, these results cannot readily be transferred to the European setting. The trial population was almost entirely Han Chinese (99%) and HBV infection was the predominant etiology (76% of patients), whereas only 5% had MASLD-related HCC. Given the influence of etiology on both tumor biology and patient characteristics discussed above, the efficacy and safety of this perioperative regimen in MASLD- and alcohol-related HCC that increasingly account for resectable disease in Europe remain essentially untested. Dedicated studies enrolling European populations, and the routine reporting of etiology-specific outcomes, are therefore needed before perioperative immunotherapy can be adopted in this setting.

### Management of intermediate-advanced stage HCC

The shift in HCC etiology towards MASLD is accompanied by an increasing burden of obesity, diabetes, hypertension, and cardiovascular disease, all of which may influence optimal treatment choices in intermediate-advanced HCC. In particular, significant cardiovascular risk profiles may affect the safety of therapies targeting the vascular endothelial growth factor (VEGF) pathway, such as bevacizumab or multityrosine-kinase inhibitors, which are associated with hypertension, thromboembolic events, cardiac complications, and an increased bleeding risk.^65^^,^^66^ Thus, in patients with relevant cardiovascular comorbidities, dual immune checkpoint blockade with programmed cell death protein 1 (PD-1) or programmed death-ligand 1 (PD-L1) inhibitors combined with cytotoxic T-lymphocyte–associated protein 4 (CTLA-4) blockers might be the regimen of choice.^67^ In addition, the age of patients diagnosed with HCC in Europe is increasing,^3^ resulting in a population with greater frailty and multimorbidity affecting eligibility for treatment. On the other hand, the more preserved hepatic function observed in MASLD-associated HCC may facilitate the (repeated) use of locoregional therapies.

Increasing attention has been directed towards the potential influence of HCC etiology on response to systemic therapies. Preclinical models of HCC arising in the context of MASH have identified dysfunctional, activated PD-1-expressing T cells that may promote hepatocarcinogenesis while potentially impairing antitumor immune responses.^68^ These findings have raised the hypothesis that the growing proportion of MASLD-related HCC could influence the effectiveness of immunotherapy at the population level. However, evidence from clinical trials has not consistently confirmed a clinically meaningful association between liver disease etiology and the efficacy of immune checkpoint inhibitors.^69^ Accordingly, despite ongoing biological interest, the underlying liver disease etiology is currently not used to guide patient selection for immune checkpoint inhibitor-based therapy in clinical practice.^11^

## Benefits of etiological treatment for HCC risk and management

Patients with an ongoing liver disease (i.e., untreated viral hepatitis or MASLD) have a higher incidence of HCC than individuals whose liver disease has been cured or effectively suppressed.^70^ Successful treatment of the underlying liver disease can lead to regression of fibrosis and even cirrhosis—the main risk factor for HCC—which has been particularly well documented in chronic viral hepatitis,^71^^,^^72^ and is associated with a reduced risk of HCC.^73^

For instance, in a five-year open-label follow-up study including patients with chronic HBV receiving tenofovir, 176 (51%) of 348 patients with biopsies at both baseline and week 240 showed regression of fibrosis, and cirrhosis resolved in 71 (74%) of 96 patients with cirrhosis at baseline.^71^ According to a systematic review, viral suppression in chronic HBV and sustained virological response (SVR) in chronic HCV were associated with histological regression of cirrhosis in 66% (range, 62–74%) and 56% (range, 24–83%) of patients, respectively.^72^ More recently, emerging treatments for MASH and primary sclerosing cholangitis achieved higher fibrosis improvement rates on histology compared with their respective placebo control arms,^74^^,^^75^ but their long-term effects are yet unknown.

Since portal hypertension is linked to liver fibrosis, etiological treatment that regresses fibrosis ameliorates portal hypertension, reduces the risk of decompensation, and promotes recompensation in patients with decompensated cirrhosis (Fig. 3A).^76^^,^^77^ Recompensation is clinically defined as resolution of any form of decompensation even after discontinuation of specific therapy (i.e., off diuretics, lactulose and rifaximin),^78^ and stable improvement of liver function following the removal of the underlying etiological trigger (cure or suppression) (Fig. 3B). According to a recent meta-analysis, etiological cure or control resulted in recompensation in approximately one-third of patients with decompensated cirrhosis, with the highest prevalence being observed in patients with viral etiologies. Recompensation is associated with a reduced risk of HCC and mortality.^77^

**Fig. 3:**
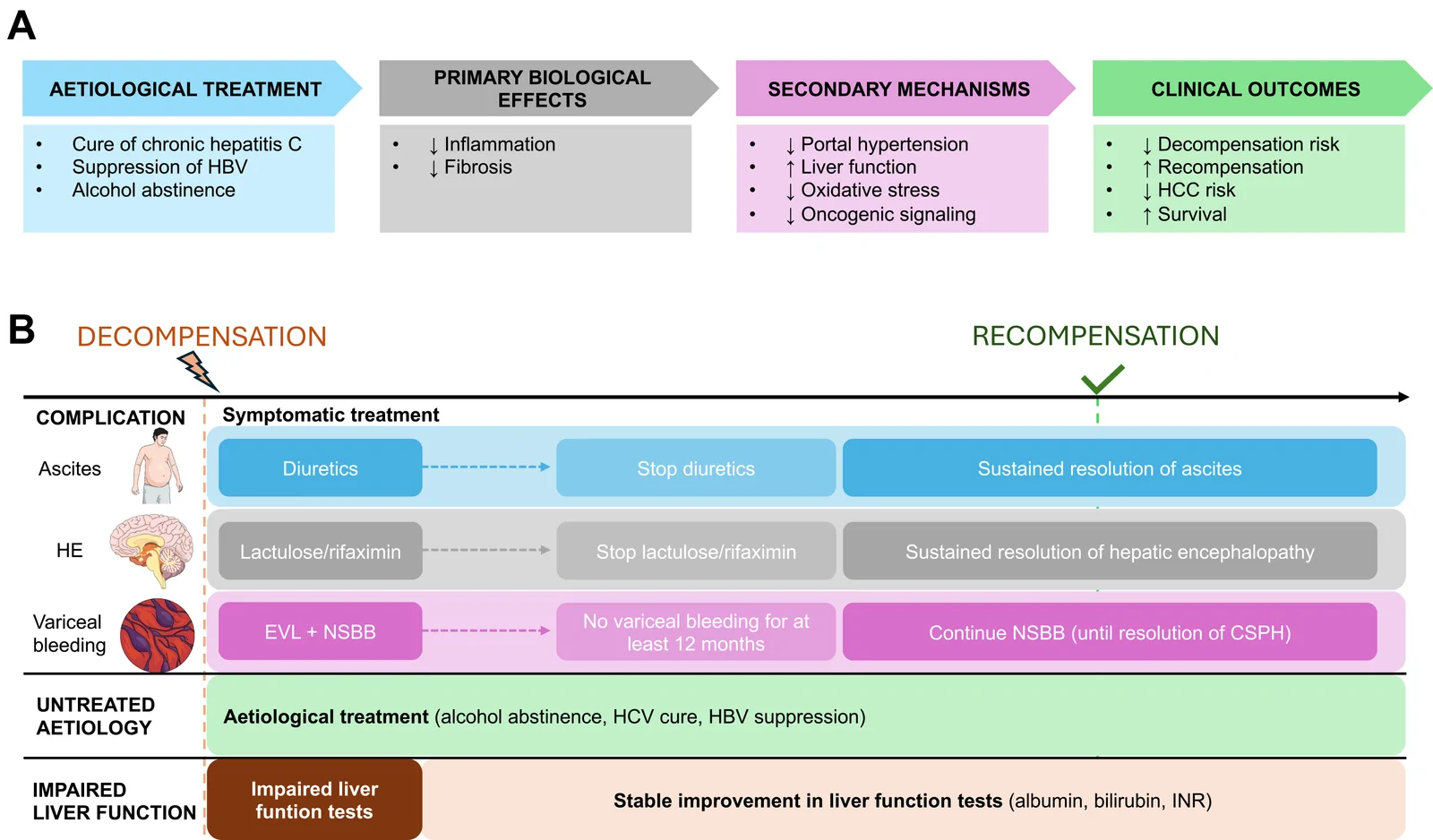
**Potential benefits of etiological treatment.** A) Biological effects of etiological treatment and their influence on clinical outcomes. B) The concept of recompensation in patients with decompensated cirrhosis. The figure was created using Mind the Graph (www.mindthegraph.com). Abbreviations: CSPH, clinically significant portal hypertension; EVL, endoscopic variceal ligation; HBV, hepatitis B virus; HCC, hepatocellular carcinoma; HCV, hepatitis C virus; HE, hepatic encephalopathy; INR, International Normalized Ratio; NSBB, non-selective beta blockers.

Preserving or optimizing hepatic function by treatment of an underlying liver disease is highly relevant for the management of patients with HCC, as treatment selection and prognosis are closely related to liver function.^79^^,^^80^ Except for liver transplantation, all recommended therapeutic options for HCC require well-preserved, compensated hepatic function,^11^^,^^79^ and impaired baseline liver function and its deterioration during treatment are both associated with worse survival.^81^^,^^82^ Indeed, successful treatment of HCV during systemic therapy with atezolizumab plus bevacizumab was independently associated with reduced risk of decompensation and mortality in a recently published retrospective multicenter study.^83^ Recompensation in patients with HCC may reopen the door for systemic or even curative therapies, as demonstrated by two recently published multicenter, cohort studies.^84^^,^^85^ Beyond its beneficial effects on hepatic function, etiological treatment may also reduce the risk of recurrence in patients with HCC undergoing curative therapy.^86^ This effect likely reflects a reduction in late recurrence, which is related to the underlying liver disease rather than true metastasis.^87^

## Public health initiatives and policies to prevent liver disease and its progression

A large proportion of liver disease and liver cancer is preventable through coordinated public health action^10^ targeting preventable or treatable risk factors.^4^ There is a growing shift in the focus from the current emphasis on the management of end-stage liver disease complications to prevention, pro-active case-finding, early identification of progressive liver fibrosis, and the timely initiation of treatment.^10^^,^^24^ However, liver disease is frequently asymptomatic until end-stage liver disease has developed, and is generally overlooked at the political level and in resource allocation, as compared to other non-communicable diseases.^6^^,^^10^ This leads to substantial gaps in the implementation of preventive interventions and population-level policies that should mitigate the social, commercial, and environmental determinants of health that drive key causes of liver disease (Table 1).^4^^,^^10^ Moreover, marked inequities persist across the prevention and care continuums, disproportionately affecting vulnerable and marginalized populations exposed to liver cancer risk factors.^4^^,^^6^ Other barriers include heterogeneity in healthcare systems across countries, competing healthcare priorities, and the limited availability of multidisciplinary care. The availability of diagnostic tests and effective treatments also influences the successful implementation of clinical care pathways. Furthermore, delivering multidisciplinary care remains challenging, as access to specialists from different disciplines within the same healthcare setting is often limited, resource-intensive, and constrained by inadequate reimbursement for services such as dietary counseling and exercise interventions.^24^^,^^88^ Early detection of progressive liver disease requires moving away from traditional, fragmented hepatology services towards integrated care models that embed both liver disease prevention and management within broader health-care delivery that focuses on common risk factors.^24^ In addition, there is variability across Europe in different primary care health system structures and financing models, and in the way they play a coordinating role in health promotion and secondary prevention or early treatment of chronic disease. Integrating chronic liver disease treatment into this coordinating role is currently poorly established in most global settings, in contrast to the established primary care-led management of other chronic diseases. Optimizing primary care management in prevention and early detection of liver disease, and establishing defined detection and management pathways across the primary and secondary care interface, needs to be a key part of tackling the increasing burden of liver disease. Seeing chronic liver disease as part of multimorbidity management in patients who are marginalized and those with complex health and social care needs should also be highlighted as key to good holistic primary care.^89^

**Table 1:** Etiology-specific public health interventions and policies, and potential barriers for implementation.

Risk factor	Public health and policy interventions	Potential barriers for implementation
Cross-cutting	• A top-down, government-led multisectoral collaboration on liver cancer prevention • Sustained political commitment and financing for prevention programs • Health education in the community • Implementing the WHO Best Buys • Integrated liver care, use of digital health systems and registries for surveillance and monitoring • Risk-stratified HCC surveillance • Policies addressing health inequities and access to care • Workforce training and capacity building in hepatology and public health • Engagement of clinicians and patient organizations, to support effective public health policies	• Liver disease often remains asymptomatic until advanced stages, which contributes to late diagnosis • Liver disease is frequently overlooked by policy-makers, as compared to other NCDs • Low public awareness of liver disease risk factors • Insufficient funding and resource allocation • Weak implementation of population-level preventive policies • Social, commercial, and environmental barriers • Structural, cultural, and legal barriers affecting migrants and marginalized communities • Limited training of healthcare workers in liver disease prevention and management
Hepatitis B	• Universal HBV vaccination, including birth-dose vaccination • Maternal HBV screening during pregnancy and neonatal prophylaxis • Expanded population-based HBV screening, particularly in high-risk groups • Improved linkage to care and long-term monitoring • Increased access to effective antiviral therapies • Integration of HBV testing within primary care and community health services • Community outreach and targeted programs for migrant populations • Strengthening surveillance systems for HBV infection • Public awareness campaigns to reduce stigma and increase testing	• Low vaccination coverage in some regions and countries • Limited HBV screening uptake • Poor linkage to care after diagnosis • Cost and limited availability of antiviral therapy in some settings • Stigma and discrimination associated with HBV infection • Barriers faced by migrants and underserved populations
Hepatitis C	• Expanded HCV screening, including birth-cohort and risk-based testing • Broad access to direct-acting antiviral treatment • Simplified diagnostic algorithms (e.g., reflex testing) • Integration of HCV services into primary care, addiction services, and prisons • Harm-reduction strategies (needle-syringe programs, opioid substitution therapy) • Community-based testing and decentralized treatment models • National HCV elimination programs and strategies aligned with WHO targets	• High cost of treatment in some countries • Limited access to screening and diagnosis • Stigma towards high-risk populations • Weak linkage to care after diagnosis • Fragmented healthcare services
Hepatitis D	• Systematic HDV testing of all individuals with chronic HBV infection • Improved epidemiological surveillance for HDV prevalence • Expanded access to novel antiviral therapies where available • Education of healthcare providers regarding HDV diagnosis and management • Integration of HDV screening within HBV care programs	• Socioeconomic barriers among high-risk populations (e.g., HBV-infected migrants and people who inject drugs) • Low awareness among clinicians and patients • Limited access to HDV diagnostic testing • Limited availability of effective treatments
MASLD	• Recognize steatotic liver disease as a preventable NCD within the core NCD framework^a^ • Expand access to lifestyle modification programs • Implement population-level nutrition policies (e.g., taxation of sugar-sweetened beverages, front-of-pack labeling, food reformulation, subsidizing fresh and minimally processed foods) • Integrating routine food insecurity screening in clinical care • Local and national policy to provide population-level physical activity opportunities and safe infrastructure • National campaigns promoting culturally tailored healthy diets • School-based healthy lifestyle interventions, with parental involvement • “Food is Medicine” healthcare programs for food-insecure patients • Social prescribing to connect individuals with nonmedical health-promoting resources (e.g., nutrition education and healthy cooking sessions, physical activity groups, etc.)	• WHO Best Buys do not address liver health specifically • Industry resistance (deny, delay, doubt, divide) • Lobbying of policy-makers by the food industry, leading to a lack of political will • Public health campaigns cannot match the aggressive marketing and resources of industry • Disproportionate marketing and availability of low-cost, unhealthy ultra-processed food and beverages • High cost of health-promoting programs and difficulties with sustainability • Insufficient health insurance coverage for nutritional treatment and limited public healthcare resources to manage a highly prevalent liver disease
Alcohol and tobacco	• Expand alcohol and tobacco cessation programs • Policy regulations on alcohol and tobacco access and marketing • Mandatory warning labels including risk of cancer and liver disease • Strengthen collaboration among health organizations to support alcohol harm reduction (e.g., European Health Alliance on Alcohol^b^) and implement the WHO-recommended tobacco and alcohol control measures	• Industry lobbying of policy-makers, scientists and advocacy groups, leading to reduced political action • Heavy marketing, including digital and weak enforcement of restrictions • Marketing and sale of alcohol to children and adolescents, especially on social media and unsupervised purchases online • Easy access to tobacco and alcohol in many countries

**Abbreviations:** HBV, hepatitis B virus; HCC, hepatocellular carcinoma; HCV, hepatitis C virus; HDV, hepatitis D virus; MASLD, metabolic dysfunction-associated steatotic liver disease; NCD, noncommunicable disease; WHO, World Health Organization.

a See the WHO draft resolution: “Liver Disease—A Missing Piece in the Global Noncommunicable Disease Response” https://apps.who.int/gb/ebwha/pdf_files/EB158/B158_CONF7-en.pdf.

b See the European Health Alliance on Alcohol (EHAA) website for more information https://alliance-ehaa.org/.

While universal HBV vaccination is the most effective intervention to reduce HCC burden, coverage in Europe remains incomplete, particularly among at-risk adult populations, because payers underestimate long-term benefits relative to upfront costs and underinvest in screening and awareness campaigns as well as primary-care training,^24^ resulting in preventable deaths.^10^ As previously mentioned, in Western and Northern Europe, chronic HBV disproportionately affects migrants from intermediate- and high-prevalence regions, who often face structural, linguistic, and legal barriers to timely diagnosis and long-term care.^90^ HCV transmission is concentrated mainly among people who inject drugs, for whom stigma, criminalization, and fragmented services limit access to harm reduction and curative therapy.^91^ The *Action Plan for the Health Sector Response to Viral Hepatitis in the WHO European Region* outlines the pathway to eliminate HBV and HCV as public health threats by 2030 through universal vaccination, expanded testing, linkage to care, and integrated service delivery.^92^ Achieving these targets will require sustained political commitment, decentralized care models, equity-oriented policies, and the implementation of integrated liver care with risk-stratified HCC surveillance.

Obesity has been repeatedly demonstrated to be an independent risk factor for HCC.^93^ Additionally, an unhealthy dietary composition characterized by high saturated fat,^94^ red and processed meat,^95^ high sugar or sugar-sweetened beverages,^96^ has also been suggested to be related to an increased HCC risk, although with varying levels of evidence. In contrast, a healthy dietary pattern like the Mediterranean diet, as well as regular physical activity are associated with reduced risk of HCC.^97^^,^^98^ The World Cancer Research Fund/American Institute for Cancer Research Expert Report, last updated in 2018, found strong evidence that alcohol consumption and body fatness increase risk while coffee consumption decreases risk for liver cancer, but there was less convincing evidence for other foods.^99^

Therefore, policies that address the reduction of exposure to unhealthy food and alcohol, while increasing the availability and affordability of healthy foods (including among those with food insecurity), and physical activity are relevant for liver disease and HCC prevention.^6^^,^^100^ The WHO best buys—policies that are evidence-based and cost-effective—are also highly relevant for liver disease prevention,^101^ but continue not to name liver health, e.g., MASLD or MASH, specifically.^102^

To combat the high and increasing consumption of alcohol, the presence of country-wide public health interventions, such as national action plans, minimum unit pricing (MUP), and restricted access, has yielded promising outcomes in reducing alcohol-related HCC incidence.^4^ For example, an ecological multi-national study demonstrated that alcohol-related public health policies were inversely associated with alcohol use disorder prevalence, alcohol-related liver disease mortality, and alcohol-attributable HCC.^103^ The HEPAHEALTH II microsimulation modeling study found that the most effective policy scenario to reduce liver cancer burden would be implementing an MUP of €1, followed by a lower MUP of €0.70 combined with a volumetric alcohol tax, and a sugar-sweetened beverage tax.^104^ Furthermore, alcohol labeling, especially cancer risk warning, has been shown to have the potential to decrease consumption,^105^ but this simple measure of mandatory label warnings about the link between alcohol and cancer and alcohol's role in liver disease has not yet been implemented.^106^ The WHO has developed a campaign to decrease harmful alcohol use, the SAFER Initiative: Strengthen restrictions on availability, Advance and enforce drink driving counter measures, Facilitate access to screening, brief interventions and treatment, Enforce bans or comprehensive restrictions on alcohol advertising, sponsorship, and promotion, Raise prices on alcohol through excise taxes and pricing policies.^107^ In addition, the WHO Regional Office for Europe developed a practical tool for advocacy—the “*Alcohol policy playbook”* designed for policy-makers, advocates, and public health professionals.^108^ Although there is a wide agreement among experts in the field about which measures are the most effective at reducing alcohol use,^109^ effective lobbying of policy-makers by the alcohol industry and a lack of political will are barriers to the implementation of these measures.

There are challenges in the implementation of healthy lifestyle habits needed for HCC prevention; access to high-quality nutrition is largely dictated by the immediate food environment, which is often driven by systemic inequity.^110^, ^111^, ^112^, ^113^ Ultra-processed food (UPF) and drinks (e.g., sugar-sweetened beverages) consumption varies across 22 European countries, with a share of dietary energy originating from UPFs ranging from 14 to 44%,^114^ with some having increased consumption over time, such as Spain and the UK.^114^, ^115^, ^116^ UPFs are usually characterized by high energy, salt, sugars, and saturated fat, but low in fiber content. Together, these foods contribute to a diet that contrasts with the Mediterranean dietary pattern,^98^ and may increase the risk for HCC, although evidence for a direct association remains scarce and conflicting.^96^ However, UPF is related to HCC risk factors, including obesity, diabetes, MASLD and advanced liver disease.^115^, ^116^, ^117^, ^118^, ^119^ Therefore, it is recommended to minimize the consumption of UPF in several clinical guidelines, including those for MASLD,^120^ cardiovascular disease,^121^ and cancer.^122^ Ultra-processed food consumption is structurally and commercially driven.^24^ Thus, Lancet series papers called for policies aiming to change the food environment by food reformulation and mandatory regulations that address and restrict the marketing, availability, and affordability of UPFs, while promoting dietary patterns based on fresh and minimally processed foods across the population, and providing targeted support for lower-income households, who face disproportionate financial and environmental barriers to accessing healthy diets.^123^^,^^124^ To tackle food insecurity, which is related to higher consumption of low-cost UPFs and lower consumption of the more costly minimally processed or unprocessed foods,^100^ the “Food is Medicine” approach^125^ and social prescribing can be used.^100^^,^^126^

Tobacco smoking is an established risk factor for HCC, with evidence that quitting smoking for more than 30 years reduces risk to levels approaching those of never smokers.^127^ Policies and public health measures recommended by the WHO should be promoted, including increased taxation, additional marketing restrictions, front-of-package labeling, large graphic health warnings on all tobacco packages, mass media campaigns, and cost-covered support for tobacco cessation.^128^

## Conclusion

The number of new liver cancer cases has been estimated to increase in Europe year on year. While the proportion of viral-related HCC is declining, the incidence of MASLD-related HCC is expected to rise and the proportion of alcohol-associated HCC recently remained stable at a very high level. According to recent estimates, an annual reduction of at least 2% in the age-standardized incidence rate (ASIR) is required to halt the increasing number of liver cancer cases globally.^4^ Considering that a large proportion of HCCs could be prevented by treating and managing modifiable risk factors, achieving this goal requires public health and policy efforts coordinated at the European Union and WHO European region levels, which target the main risk factors for HCC, including viral hepatitis, alcohol, and metabolic conditions. Treatment of the underlying etiological cause and avoidance of other risk factors not only decrease the risk of HCC, but also benefits patients who have already developed HCC. Etiological treatment can reverse liver fibrosis, improve portal hypertension, decrease the risk of decompensation, and even promote recompensation in patients with decompensated cirrhosis. This can help to preserve or even improve liver function, which is of utmost importance for the management of patients with HCC, as both treatment decisions and prognosis are closely related to liver function. Fig. 4 summarizes the epidemiological and etiological trends of HCC and their implications for clinical presentation at diagnosis and for the management of HCC across different disease stages.

**Key recommendations**

• Encourage coordinated European HCC prevention strategies focusing on the detection and control of viral hepatitis, reduction of alcohol-related harm, improvement of metabolic health and tobacco control.
• Improve alcohol-control policies, including minimum unit pricing, adequate taxation, restrictions on availability and marketing, cancer-warning labels and access to screening and treatment for alcohol use disorder.
• Improve metabolic health at the population-level by taxation of unhealthy foods and drinks, reducing the availability and marketing of unhealthy/ultra-processed foods while improving access to healthy, minimally processed foods and physical activity.
• Implement liver health pathways in primary care including screening for the presence of liver fibrosis, metabolic risk management, testing for viral hepatitis and establishment of referral networks.
• Prioritize evidence-based HCC surveillance programmes; surveillance in high-risk patients with non-cirrhotic MASLD should be individualized, and research should be promoted to inform the development of structured surveillance programmes.
• Integrate liver health explicitly into European health policies, with sustainable funding for prevention, screening and multidisciplinary care of liver cancer.
• Strengthen the European evidence base for emerging treatment strategies such as multidisciplinary pre-habilitation and neoadjuvant immunotherapy prior to surgical therapies in patients with HCC.

**Search strategy and selection criteria**

References for this Series paper were identified through searches of PubMed with the search terms “hepatocellular carcinoma”, “liver cancer “, “epidemiology”, “migration”, “etiology”, “fibrosis”, “cirrhosis”, “decompensation”, “recompensation”, “viral hepatitis“, “hepatitis B virus”, “hepatitis C virus”, “hepatitis delta virus”, “MASLD”, “MASH”, “alcohol-related liver disease”, “prevention”, “surveillance”, “treatment”, “public health”, “policy”, “smoking”, “lifestyle”, “diet” until March 2026. Articles were also identified through searches of the authors’ own files. Only papers published in English were reviewed. The final reference list was generated on the basis of originality and relevance to the broad scope of this Series paper.

**Fig. 4:**
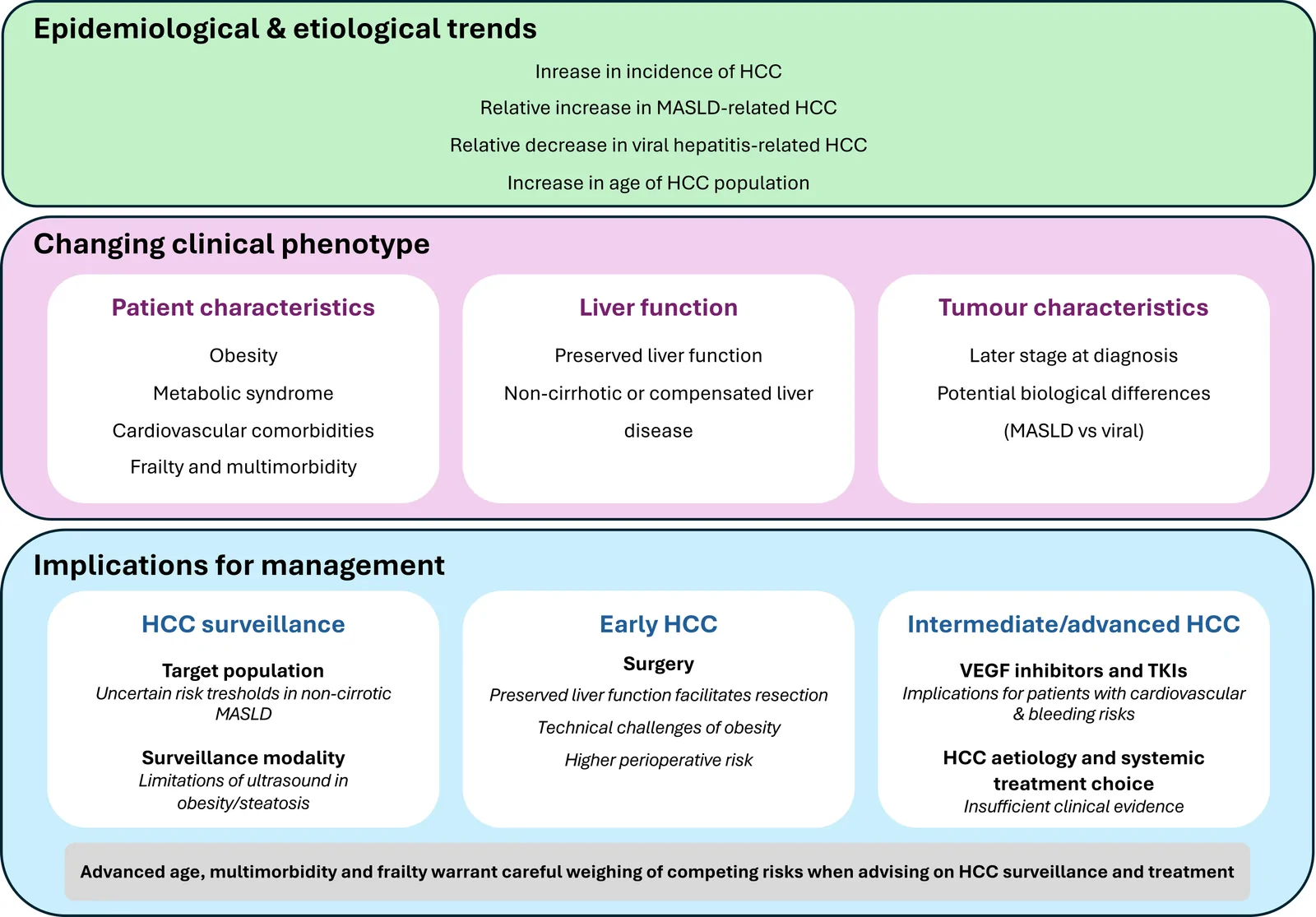
**Clinical implications of the evolving epidemiological and etiological landscape of hepatocellular carcinoma in Europe.** Abbreviations: HCC, hepatocellular carcinoma; MASLD, metabolic dysfunction-associated steatotic liver disease; VEGF, vascular endothelial growth factor; TKIs, tyrosine kinase inhibitors.

## Contributors

All authors contributed to conceptualization, methodology, literature review, writing of the original draft, and reviewing and editing.

## Editor note

The Lancet Group takes a neutral position with respect to territorial claims in published maps and institutional affiliations.

## Declaration of interests

BS received grant support from AstraZeneca, Eisai, Ipsen and Roche Diagnostics, speaker honoraria from AstraZeneca, BMS and Eisai as well as travel support from AbbVie, AstraZeneca, Ipsen, Gilead and Roche.

JD received research support from AstraZeneca, Bayer and Roche, speaker honoraria from Amgen, Astellas, AstraZeneca, Bayer, BeOne, BMS, Eisai, Ipsen, Lilly, Merck, MSD, Roche and Servier, consulting fees from AbbVie, Astellas, AstraZeneca, Bayer, BeOne, BMS, Eisai, Elmidix, Medtalks, Merck, MSD, Novartis, Incyte, Ipsen, Roche and Takeda as well as travel support from Amgen, AstraZeneca, Ipsen, Servier and Roche.

FP received honoraria from Astrazeneca, BMS, EISAI, MSD, Nerviano, NovoNordisk, Roche, Siemens Healthineers to attend advisory boards, from Astrazeneca, Bracco, ESAOTE, EISAI, GE, Gilead, IPSEN, MSD, Roche, Samsung, Siemens Healthineers for lecturing, from Bracco, Nerviano, Signant Health as consultant and from Roche for attending congresses.

AV received honoraria from Astrazeneca, and Roche as consultant and from Astrazeneca for attending congresses.

LAK received research grants to her Institution by Gilead Sciences outside of the submitted paper.

BP received speaker honoraria from AbbVie, Takeda, Gilead, Opella Healthcare and consulting fees from Boehringer Ingelheim.

JVL received grants to his institutions from Boehringer Ingelheim, Echosens, Gilead Sciences, Madrigal Pharmaceuticals, MSD, Novo Nordisk, and Pfizer, consulting fees from Echosens, GSK, Madrigal Pharmaceuticals, Novo Nordisk, Pfizer and Takeda, a paid leadership role at the Global NASH Council (ended), and honoraria for lectures from Echosens, Gilead Sciences, GSK, MSD, Novo Nordisk, and Pfizer, outside of the submitted work.

MB received research support from Gilead, speaker honoraria from Abbvie, GSK, Gilead; Janssen and Rocha.

SZS is a member of the European Association for the Study of the Liver (EASL) governing board (medical association) and a researcher (paid) at the Global MASH council (research body in the US).

MP received speaker honoraria from AstraZeneca, Bayer, BMS, Eisai, Ipsen, Lilly, MSD, and Roche; he is a consultant/advisory board member for AstraZeneca, Bayer, BMS, Eisai, Ipsen, Lilly, MSD, and Roche; he received grants from AstraZeneca, Bayer, BMS, Eisai, and Roche; he received travel support from AstraZeneca, Bayer, BMS, Ipsen, and Roche. He is an independent data monitoring committee member for CatalYm. He is a member of the European Association for the Study of the Liver (EASL) governing board (medical association).

## References

[1] Bray F., Laversanne M., Sung H. (2024). Global cancer statistics 2022: GLOBOCAN estimates of incidence and mortality worldwide for 36 cancers in 185 countries. CA Cancer J Clin.

[2] European Cancer Information System (ECIS) (2024). Liver cancer in the EU. https://ecis.jrc.ec.europa.eu/sites/default/files/2024-10/2024-10-03_Factsheet_Liver-Cancer.pdf.

[3] Tan E.Y., Danpanichkul P., Yong J.N. (2025). Liver cancer in 2021: Global Burden of Disease study. J Hepatol.

[4] Chan S.L., Sun H.C., Xu Y. (2025). The Lancet Commission on addressing the global hepatocellular carcinoma burden: comprehensive strategies from prevention to treatment. Lancet.

[5] Wedemeyer H., Tergast T.L., Lazarus J.V. (2023). Securing wider EU commitment to the elimination of hepatitis C virus. Liver Int.

[6] Kondili L.A., Lazarus J.V., Jepsen P. (2024). Inequities in primary liver cancer in Europe: the state of play. J Hepatol.

[7] Tan D.J.H., Ng C.H., Lin S.Y. (2022). Clinical characteristics, surveillance, treatment allocation, and outcomes of non-alcoholic fatty liver disease-related hepatocellular carcinoma: a systematic review and meta-analysis. Lancet Oncol.

[8] Ferlay J., Ervik M., Lam F. (2024). Global cancer observatory: Cancer today (version 1.1). https://gco.iarc.who.int/today.

[9] Kazi O., Hamidzadeh S., Ehsani Zunuz B. (2025). Burden of liver cancer in the European region from 1990 to 2021: a sub-analysis of the global burden of disease study. Ann Med Surg.

[10] Karlsen T.H., Sheron N., Zelber-Sagi S. (2022). The EASL-Lancet Liver Commission: protecting the next generation of Europeans against liver disease complications and premature mortality. Lancet.

[11] (2025). EASL Clinical Practice Guidelines on the management of hepatocellular carcinoma. J Hepatol.

[12] Vaz J., Jepsen P., Stromberg U. (2025). Metabolic dysfunction-associated steatotic liver disease has become the most common cause of hepatocellular carcinoma in Sweden: a nationwide cohort study. Int J Cancer.

[13] van Son K.C., van Eekhout K.M.A., de Vos-Geelen J. (2026). Increasing incidence and changing trends in etiology & survival rates in HCC in a European cohort. JHEP Rep.

[14] Berenguer P.H., Fraga C., Müller S. (2026). Etiologic profile and prognostic patterns of hepatocellular carcinoma in a Southern European population - madeira, Portugal: insight into a preventable cancer. Cancer Epidemiol.

[15] Crişan D., Procopeț B., Crăciun R. (2025). The role of easy-to-use non-invasive scores in the assessment of hepatocellular carcinoma prognosis - data from the Romanian hepatocellular carcinoma registry. J Gastrointestin Liver Dis.

[16] Skladaný Ľ., Adamcová-Selčanová S., Malec V. (2018). Hepatocellular carcinoma in central Slovakia – tertiary referral centre experience with 207 patients. Gastroent Hepatol.

[17] Pasta A., Pieri G., Giannini E.G., Italian Liver Cancer ITA.LI.CA Group (2025). Changing epidemiology of hepatocellular carcinoma in the Mediterranean area: a challenge for surveillance?. JHEP Rep.

[18] Sala M., Pascual S., Rota Roca M.R. (2025). Evolving epidemiology of HCC in Spain. JHEP Rep.

[19] Hucke F., Emmer H., Emmer R. (2023). Changes in the epidemiology of hepatocellular carcinoma in Carinthia, Austria, 2012-2023. Cancers (Basel).

[20] Schrodi S., Berger-Thurmel K., Becht S. (2025). Treatment gaps and survival outcomes of hepatocellular carcinoma: insights from a nationwide, claims-based study. Ther Adv Med Oncol.

[21] Nguyen-Khac E., Merle P., Ben Khadhra H. (2025). Real-life data on hepatocellular carcinoma in the French prospective CHIEF cohort in the era of immunotherapy. Eur J Gastroenterol Hepatol.

[22] Burton A., Balachandrakumar V.K., Driver R.J. (2022). Regional variations in hepatocellular carcinoma incidence, routes to diagnosis, treatment and survival in England. Br J Cancer.

[23] Liu Y., Zheng J., Hao J. (2022). Global burden of primary liver cancer by five etiologies and global prediction by 2035 based on global burden of disease study 2019. Cancer Med.

[24] Karlsen T.H., Hutchinson S.J., Zelber-Sagi S. (2026). Implementing sustainable liver health in Europe: a second EASL-Lancet Commission. Lancet.

[25] European Centre for Disease Prevention and Control (2025).

[26] Patmore L.A., Carey I., Feld J.J. (2026). Prediction of hepatocellular carcinoma and other liver-related events in chronic hepatitis B patients with metabolic dysfunction or metabolic dysfunction-associated steatotic liver disease. Aliment Pharmacol Ther.

[27] Simao M., Goncalves C. (2024). Hepatitis C virus infection in Europe. Pathogens.

[28] Demirel A., Uraz S., Deniz Z. (2024). Epidemiology of hepatitis D virus infection in Europe: is it vanishing?. J Viral Hepat.

[29] Huang D.Q., Mathurin P., Cortez-Pinto H., Loomba R. (2023). Global epidemiology of alcohol-associated cirrhosis and HCC: trends, projections and risk factors. Nat Rev Gastroenterol Hepatol.

[30] Khan S.A., Vithayathil M., Yip T.C.F., El-Serag H.B. (2026). The changing epidemiology of primary liver cancer in the 21st century. J Hepatol.

[31] Haldar D., Kern B., Hodson J. (2019). Outcomes of liver transplantation for non-alcoholic steatohepatitis: a European Liver Transplant Registry study. J Hepatol.

[32] Goff C., Shaikh A., Goli K. (2023). Contemporary changes in etiology for hepatocellular carcinoma in liver transplantation. Clin Gastroenterol Hepatol.

[33] Farah M., Anugwom C., Ferrer J.D. (2023). Changing epidemiology of hepatocellular carcinoma in South America: a report from the South American liver research network. Ann Hepatol.

[34] Takeuchi Y., Tateishi R., Obi S. (2026). Temporal trends and regional variations in hepatocellular carcinoma etiology: a multinational study across Asia. Hepatol Int.

[35] Eurostat (2025). EU population diversity by citizenship and country of birth. https://ec.europa.eu/eurostat/statistics-explained/index.php?title=EU_population_diversity_by_citizenship_and_country_of_birth.

[36] European Centre for Disease Prevention and Control (2016).

[37] Razavi-Shearer D.M., Kondili L.A., Hall S. (2025). Estimating the impact of displacement from Ukraine on HBV and HCV prevalence among migrants in the European Union, 2024: a modeling study. Lancet Reg Health Eur.

[38] Kim J.U., Ingiliz P., Shimakawa Y., Lemoine M. (2021). Improving care of migrants is key for viral hepatitis elimination in Europe. Bull World Health Organ.

[39] Patmore L.A., van Eekhout K.M.A., Buti M. (2024). Hepatocellular carcinoma risk in sub-Saharan African and Afro-Surinamese individuals with chronic hepatitis B living in Europe. J Hepatol.

[40] Toh M.R., Wong E.Y.T., Wong S.H. (2023). Global epidemiology and genetics of hepatocellular carcinoma. Gastroenterology.

[41] Abed Al Ahad M., Fenyk M. (2026). Obesity and the experienced cultural, economic, and climate variations among immigrants to high-income countries: an umbrella review of the literature. Public Health Nutr.

[42] Meeks K.A., Freitas-Da-Silva D., Adeyemo A. (2016). Disparities in type 2 diabetes prevalence among ethnic minority groups resident in Europe: a systematic review and meta-analysis. Intern Emerg Med.

[43] Duberg A.S., Lybeck C., Fält A., Montgomery S., Aleman S. (2022). Chronic hepatitis B virus infection and the risk of hepatocellular carcinoma by age and country of origin in people living in Sweden: a national register study. Hepatol Commun.

[44] Baggaley R.F., Zenner D., Bird P. (2022). Prevention and treatment of infectious diseases in migrants in Europe in the era of universal health coverage. Lancet Public Health.

[45] Castellana M., Donghia R., Lampignano L. (2021). Prevalence of the absence of cirrhosis in subjects with NAFLD-associated hepatocellular carcinoma. J Clin Med.

[46] Burke L., Hinkson A., Haghnejad V., Jones R., Parker R., Rowe I.A. (2025). Hepatocellular carcinoma risk scores for non-viral liver disease: a systematic review and meta-analysis. JHEP Rep.

[47] Indre M.G., Stefanini B., Boe M. (2026). HCC is the predominant liver-related event in MASLD: 2-Step non-invasive algorithms to stratify risk in non-cirrhotic patients. J Hepatocell Carcinoma.

[48] Sanyal A.J., Van Natta M.L., Clark J. (2021). Prospective study of outcomes in adults with nonalcoholic Fatty liver disease. N Engl J Med.

[49] Chhatwal J., Hajjar A., Mueller P.P. (2024). Hepatocellular carcinoma incidence threshold for surveillance in virologically cured hepatitis C individuals. Clin Gastroenterol Hepatol.

[50] Toyoda H., Kanneganti M., Melendez-Torres J. (2024). Regional differences in clinical presentation and prognosis of patients with post-sustained virologic response hepatocellular carcinoma. Clin Gastroenterol Hepatol.

[51] Fan R., Papatheodoridis G., Sun J. (2020). aMAP risk score predicts hepatocellular carcinoma development in patients with chronic hepatitis. J Hepatol.

[52] King M.J., Lee K.M., Rosberger S. (2023). HCC screening with ultrasound: assessment of quality using ultrasound LI-RADS score. Abdom Radiol.

[53] Ioannou G.N., Taddei T.H., Planeta B.M. (2026). Practice changing RCT design and rationale: abbreviated MRI plus AFP vs. ultrasound plus AFP for HCC surveillance in cirrhosis (PREMIUM study). JHEP Rep.

[54] Berardi G., Ratti F., Sposito C. (2023). Model to predict major complications following liver resection for HCC in patients with metabolic syndrome. Hepatology.

[55] Burra P., Becchetti C., Germani G. (2020). NAFLD and liver transplantation: disease burden, current management and future challenges. JHEP Rep.

[56] Ngonadi C., Hopkins L.J., Williams F.R., Armstrong M.J. (2026). Physical frailty and Sarcopenia in liver transplantation: how should they be assessed and addressed?. Transplantation.

[57] Cabibbo G., Daniele B., Borzio M. (2024). Multidisciplinary treatment of hepatocellular carcinoma in 2023: Italian practice Treatment Guidelines of the Italian Association for the Study of the Liver (AISF), Italian Association of Medical Oncology (AIOM), Italian Association of Hepato-Bilio-Pancreatic Surgery (AICEP), Italian Association of Hospital Gastroenterologists (AIGO), Italian Association of Radiology and Clinical Oncology (AIRO), Italian Society of Pathological Anatomy and Diagnostic Cytology (SIAPeC-IAP), Italian Society of Surgery (SIC), Italian Society of Gastroenterology (SIGE), Italian Society of Medical and Interventional Radiology (SIRM), Italian Organ Transplant Society (SITO), and Association of Patients with Hepatitis and Liver Disease (EpaC) - part I - surgical treatments. Dig Liver Dis.

[58] Berardi G., Ivanics T., Sapisochin G. (2023). Minimally invasive versus open liver resections for hepatocellular carcinoma in patients with metabolic syndrome. Ann Surg.

[59] Vitale A., Trapani S., Russo F.P. (2024). Waiting list mortality and 5-year transplant survival benefit of patients with MASLD: an Italian liver transplant registry study. JHEP Rep.

[60] Lim W.H., Ng C.H., Tan D. (2023). Natural history of NASH cirrhosis in liver transplant waitlist registrants. J Hepatol.

[61] Parente A., Milana F., Hajibandeh S. (2025). Liver transplant for hepatocellular carcinoma in metabolic dysfunction-associated steatotic liver disease versus other etiologies: a meta-analysis. Dig Liver Dis.

[62] Vitale A., Svegliati-Baroni G., Ortolani A. (2023). Epidemiological trends and trajectories of MAFLD-associated hepatocellular carcinoma 2002-2033: the ITA.LI.CA database. Gut.

[63] Vigano L., Conci S., Cescon M. (2015). Liver resection for hepatocellular carcinoma in patients with metabolic syndrome: a multicenter matched analysis with HCV-related HCC. J Hepatol.

[64] Wang Z., Fan J., Zhou S. (2025). Perioperative camrelizumab plus rivoceranib versus surgery alone in patients with resectable hepatocellular carcinoma at intermediate or high risk of recurrence (CARES-009): a randomised phase 2/3 trial. Lancet.

[65] Vallerio P., Orenti A., Tosi F. (2022). Major adverse cardiovascular events associated with VEGF-targeted anticancer tyrosine kinase inhibitors: a real-life study and proposed algorithm for proactive management. ESMO Open.

[66] Niimura T., Goda M., Miyata K. (2023). Evaluation of cardiovascular toxicity of the atezolizumab and bevacizumab combination. Front Drug Saf Regul.

[67] Peeters F., Dekervel J. (2023). Considerations for individualized first-line systemic treatment in advanced hepatocellular carcinoma. Curr Opin Pharmacol.

[68] Pfister D., Nunez N.G., Pinyol R. (2021). NASH limits anti-tumour surveillance in immunotherapy-treated HCC. Nature.

[69] Meyer T., Galani S., Lopes A., Vogel A. (2023). Aetiology of liver disease and response to immune checkpoint inhibitors: an updated meta-analysis confirms benefit in those with non-viral liver disease. J Hepatol.

[70] Singal A.G., Kanwal F., Llovet J.M. (2023). Global trends in hepatocellular carcinoma epidemiology: implications for screening, prevention and therapy. Nat Rev Clin Oncol.

[71] Marcellin P., Gane E., Buti M. (2013). Regression of cirrhosis during treatment with tenofovir disoproxil fumarate for chronic hepatitis B: a 5-year open-label follow-up study. Lancet.

[72] Manne V., Akhtar E., Saab S. (2014). Cirrhosis regression in patients with viral hepatitis B and C: a systematic review. J Clin Gastroenterol.

[73] Morgan R.L., Baack B., Smith B.D., Yartel A., Pitasi M., Falck-Ytter Y. (2013). Eradication of hepatitis C virus infection and the development of hepatocellular carcinoma: a meta-analysis of observational studies. Ann Intern Med.

[74] Trauner M., Trivedi P.J., Denk G. (2025). Norucholic acid for the treatment of primary sclerosing cholangitis: 96-week analysis of a pivotal phase 3 trial; LBO-001.

[75] Sanyal A.J., Newsome P.N., Kliers I. (2025). Phase 3 trial of semaglutide in metabolic dysfunction-associated steatohepatitis. N Engl J Med.

[76] Lens S., Baiges A., Alvarado-Tapias E. (2020). Clinical outcome and hemodynamic changes following HCV eradication with oral antiviral therapy in patients with clinically significant portal hypertension. J Hepatol.

[77] Goh X.E., Goh M.X., Chen V.L., Garcia-Tsao G., Abraldes J.G., Wong Y.J. (2026). Prevalence and clinical outcomes of recompensation in decompensated cirrhosis: a systematic review and meta-analysis. Clin Gastroenterol Hepatol.

[78] de Franchis R., Bosch J., Garcia-Tsao G., Reiberger T., Ripoll C., Baveno VII Faculty (2022). Baveno VII - Renewing consensus in portal hypertension. J Hepatol.

[79] Reig M., Sanduzzi-Zamparelli M., Forner A. (2026). BCLC strategy for prognosis prediction and treatment recommendations: the 2026 update. J Hepatol.

[80] Pinter M., Fulgenzi C.A.M., Pinato D.J., Scheiner B. (2025). Systemic treatment in patients with hepatocellular carcinoma and advanced liver dysfunction. Gut.

[81] Marrero J.A., Kudo M., Venook A.P. (2016). Observational registry of sorafenib use in clinical practice across Child-Pugh subgroups: the GIDEON study. J Hepatol.

[82] Stella L., Pallozzi M., Cerrito L. (2025). Liver decompensation in patients with hepatocellular carcinoma treated with atezolizumab plus bevacizumab: a real-life study. Clin Gastroenterol Hepatol.

[83] Stella L., Cabibbo G., Celsa C. (2025). Hepatitis C eradication improves oncologic and clinical outcomes in patients treated with atezolizumab plus bevacizumab. Liver Int.

[84] Zheng H., Liu Q., Wu N. (2026). Ablation for hepatocellular carcinoma in hepatitis B-related recompensated vs. compensated cirrhosis. Eur Radiol.

[85] Pinero F., Anders M., Bermudez C. (2025). Hepatic recompensation before systemic therapy for hepatocellular carcinoma yields comparable survival to compensated cirrhosis. Liver Int.

[86] Singal A.K., Freeman D.H., Anand B.S. (2010). Meta-analysis: interferon improves outcomes following ablation or resection of hepatocellular carcinoma. Aliment Pharmacol Ther.

[87] Imamura H., Matsuyama Y., Tanaka E. (2003). Risk factors contributing to early and late phase intrahepatic recurrence of hepatocellular carcinoma after hepatectomy. J Hepatol.

[88] Younossi Z.M., Wong V.W., Tsochatzis E.A. (2025). Managing MASLD through preventive hepatology: integrating policy reform, public health and personalised care. Liver Int.

[89] Jarvis H., Brennan P.N., Casanovas T. (2026). The role of primary care in response to the global liver disease syndemic: a Lancet Gastroenterology & Hepatology Commission. Lancet Gastroenterol Hepatol.

[90] Buti M., Duffell E. (2024). HBV elimination in Europe: current status and challenges. Clin Liver Dis.

[91] Pawlotsky J.M. (2026). Hepatitis C virus elimination: so close, so far?. Antiviral Res.

[92] World Health Organization Regional Office for Europe (2017). https://www.who.int/europe/publications/i/item/9789289052870.

[93] Sohn W., Lee H.W., Lee S. (2021). Obesity and the risk of primary liver cancer: a systematic review and meta-analysis. Clin Mol Hepatol.

[94] Yang W., Sui J., Ma Y. (2020). High dietary intake of vegetable or polyunsaturated fats is associated with reduced risk of hepatocellular carcinoma. Clin Gastroenterol Hepatol.

[95] Farvid M.S., Sidahmed E., Spence N.D., Mante Angua K., Rosner B.A., Barnett J.B. (2021). Consumption of red meat and processed meat and cancer incidence: a systematic review and meta-analysis of prospective studies. Eur J Epidemiol.

[96] Zhao L., Zhang X., Coday M. (2023). Sugar-sweetened and artificially sweetened beverages and risk of liver cancer and chronic liver disease mortality. JAMA.

[97] Baumeister S.E., Schlesinger S., Aleksandrova K. (2019). Association between physical activity and risk of hepatobiliary cancers: a multinational cohort study. J Hepatol.

[98] Turati F., Trichopoulos D., Polesel J. (2014). Mediterranean diet and hepatocellular carcinoma. J Hepatol.

[99] World Cancer Research Fund/American Institute for Cancer Research. Continuous Update Project Expert Report 2018. Diet, nutrition, physical activity and liver cancer. Available at: dietandcancerreport.org.

[100] Zelber-Sagi S., Carrieri P., Pericas J.M., Ivancovsky-Wajcman D., Younossi Z.M., Lazarus J.V. (2024). Food inequity and insecurity and MASLD: burden, challenges, and interventions. Nat Rev Gastroenterol Hepatol.

[101] Lazarus J.V., Mark H.E., Alkhouri N. (2024). Best buy interventions to address the burden of steatotic liver disease. Lancet Gastroenterol Hepatol.

[102] Lazarus J.V., Agirre-Garrido L., White T.M. (2026). Best buys for metabolic dysfunction-associated steatotic liver disease and metabolic dysfunction-associated steatohepatitis: a global Delphi study. Lancet Gastroenterol Hepatol.

[103] Diaz L.A., Fuentes-Lopez E., Idalsoaga F. (2024). Association between public health policies on alcohol and worldwide cancer, liver disease and cardiovascular disease outcomes. J Hepatol.

[104] Retat L., Webber L., Jepsen P. (2024). Preventing liver disease with policy measures to tackle alcohol consumption and obesity: the HEPAHEALTH II study. J Hepatol.

[105] Schwartz D., Torres-Ulloa I., Corvalan C. (2024). Effectiveness of alcohol warning labels for at-risk groups and the general public: a policy-informing randomized experiment in Chile. Prev Med.

[106] Murray F. (2025). On-label alcohol beverage warnings in Ireland- setting a standard for Europe. Lancet Reg Health Eur.

[107] WHO (2019). https://www.who.int/initiatives/SAFER.

[108] (2024). Empowering public health advocates to navigate alcohol policy challenges: alcohol policy playbook.

[109] Parker R., Arab J.P., Lazarus J.V., Bataller R., Singal A.K. (2025). Public health policies to prevent alcohol-related liver disease. Nat Rev Gastroenterol Hepatol.

[110] Paik J.M., Duong S., Zelber-Sagi S., Lazarus J.V., Henry L., Younossi Z.M. (2024). Food insecurity, low household income, and low education level increase the risk of having metabolic dysfunction-associated Fatty liver disease among adolescents in the United States. Am J Gastroenterol.

[111] Younossi Z.M., Zelber-Sagi S., Kuglemas C. (2025). Association of food insecurity with MASLD prevalence and liver-related mortality. J Hepatol.

[112] Paik A., Henry L., de Avila L. (2025). Food swamps and food deserts impact on metabolic dysfunction-associated steatotic liver disease mortality in US counties. Clin Gastroenterol Hepatol.

[113] Bevel M.S., Tsai M.H., Parham A., Andrzejak S.E., Jones S., Moore J.X. (2023). Association of food deserts and food swamps with obesity-related cancer mortality in the US. JAMA Oncol.

[114] Mertens E., Colizzi C., Penalvo J.L. (2022). Ultra-processed food consumption in adults across Europe. Eur J Nutr.

[115] Monteiro C.A., Louzada M.L., Steele-Martinez E. (2025). Ultra-processed foods and human health: the main thesis and the evidence. Lancet.

[116] Monteiro C.A., Cannon G., Moubarac J.C., Levy R.B., Louzada M.L.C., Jaime P.C. (2018). The UN decade of nutrition, the NOVA food classification and the trouble with ultra-processing. Public Health Nutr.

[117] Vadiveloo M.K., Gardner C.D., Bleich S.N. (2025). Ultraprocessed foods and their association with cardiometabolic health: evidence, gaps, and opportunities: a science advisory from the American Heart Association. Circulation.

[118] Grinshpan L.S., Eilat-Adar S., Ivancovsky-Wajcman D., Kariv R., Gillon-Keren M., Zelber-Sagi S. (2024). Ultra-processed food consumption and non-alcoholic fatty liver disease, metabolic syndrome and insulin resistance: a systematic review. JHEP Rep.

[119] Zhao L., Clay-Gilmour A., Zhang J., Zhang X., Steck S.E. (2024). Higher ultra-processed food intake is associated with adverse liver outcomes: a prospective cohort study of UK Biobank participants. Am J Clin Nutr.

[120] European Association for the Study of the L, European Association for the Study of D, European Association for the Study of O (2024). EASL-EASD-EASO clinical practice guidelines on the management of metabolic dysfunction-associated steatotic liver disease (MASLD). J Hepatol.

[121] Lichtenstein A.H., Appel L.J., Vadiveloo M. (2021). 2021 Dietary guidance to improve cardiovascular health: a scientific statement from the American Heart Association. Circulation.

[122] Rock C.L., Thomson C., Gansler T. (2020). American Cancer Society guideline for diet and physical activity for cancer prevention. CA Cancer J Clin.

[123] Scrinis G., Popkin B.M., Corvalan C. (2025). Policies to halt and reverse the rise in ultra-processed food production, marketing, and consumption. Lancet.

[124] Baker P., Slater S., White M. (2025). Towards unified global action on ultra-processed foods: understanding commercial determinants, countering corporate power, and mobilising a public health response. Lancet.

[125] Mozaffarian D., Blanck H.M., Garfield K.M., Wassung A., Petersen R. (2022). A food is medicine approach to achieve nutrition security and improve health. Nat Med.

[126] Ivancovsky-Wajcman D., Brennan P.N., Kopka C.J. (2023). Integrating social nutrition principles into the treatment of steatotic liver disease. Commun Med.

[127] Petrick J.L., Campbell P.T., Koshiol J. (2018). Tobacco, alcohol use and risk of hepatocellular carcinoma and intrahepatic cholangiocarcinoma: the Liver Cancer Pooling Project. Br J Cancer.

[128] https://www.who.int/news/item/26-05-2023-more-ways--to-save-more-lives--for-less-money----world-health-assembly-adopts-more-best-buys--to-tackle-noncommunicable-diseases.

